# Plausible Obesity-Related Chronometabolic and Nutrigenetic Nexus Concerning Dinner Glycemic Index and the *FAAH C385A* Variant

**DOI:** 10.3390/biom16020274

**Published:** 2026-02-09

**Authors:** Barbara Vizmanos, Alejandra Betancourt-Núñez, Erika Sierra-Ruelas, Juan José López Gómez, Daniel Rico, J. Alfredo Martínez, Daniel A. De Luis

**Affiliations:** 1 Programas de Doctorado en Cs. de la Nutrición Traslacional y de la Salud Pública, Laboratorio de Evaluación del Estado Nutricio, Centro de Investigación Educativa y Bienestar Universitario, Instituto de Nutrigenética y Nutrigenómica Traslacional, Centro Universitario de Ciencias de la Salud, Universidad de Guadalajara, Guadalajara 44340, Mexico; bvizmanos@yahoo.com.mx (B.V.); alejandra.bnunez@academicos.udg.mx (A.B.-N.); ln.erikasierra@outlook.com (E.S.-R.); 2 Centro de Investigación de Endocrinología y Nutrición Clínica, Universidad de Valladolid, 47003 Valladolid, Spain; jlopezgo@saludcastillayleon.es (J.J.L.G.); daniel.rico@uva.es (D.R.); 3 Dirección de Investigación, Secretaría de Salud Jalisco, Guadalajara 44100, Mexico; 4 Health Research Institute of Valladolid, Instituto de Investigación Biosanitaria de Valladolid (IBioVALL), 47010 Valladolid, Spain; 5 Nutrición de Precisión y Programa de Salud Cardio Metabólica, Institutos Madrileños de Estudios Avanzados Nutrición (IMDEA Nutrition), Consejo Superior de Investigaciones Científicas-Campus de Excelencia Internacional (CSIC-CEI), 28049 Madrid, Spain; 6 Centro de Investigación Biomédica en Red Fisiopatologia de la Obesidad y la Nutrición (CIBEROBN), Instituto de Salud Carlos III, 28029 Madrid, Spain

**Keywords:** glycemic index, obesity, gene–diet interaction, nutrigenomics, insulin, chrononutrition, chronometabolic, *C385A FAAH*

## Abstract

The interaction between chrono-nutrition (dinner intake), glycemic index (GI), and the *C358A* variant of the endocannabinoid-degrading enzyme fatty acid amide hydrolase (FAAH), along with its impact on morning fasting insulin and glycemia, has not been previously explored. This study provides new insights into chronometabolic and nutrigenetic interactions. This study aims to analyze the association between the dinner GI and the C385A variant in the *FAAH* gene with respect to fasting glucose, insulin levels, and HOMA-IR in adults with obesity. It was hypothesized that the dinner GI, probably influenced by the *FAAH* variant, could be associated with glycemic homeostasis in adults with obesity. This is a secondary analysis of a cross-sectional study focused on 189 adults with obesity (129 women; mean age, 41 ± 12 years; mean BMI, 38.0 ± 5.2 kg/m^2^). Dietary intake was assessed through two 24 h food records, enabling the calculation of GI and macronutrient composition at each meal, especially dinner. Fasting-parameter setting and genotyping were done during the study. The lineal regression analyses were adjusted by age, sex, BMI, energy intake and dinner protein. Participants with lower fasting glucose levels had higher total GI and dinner GI values than those with higher fasting glucose levels, whereas no differences in dinner GI were observed across groups stratified by insulin or HOMA-IR levels. In fully adjusted regression models, dinner GI values remained inversely associated with fasting glucose levels (β = −0.172, 95%CI −0.298 to −0.045; *p* = 0.008). The *FAAH* C385A variant independently predicted lower insulin (β = −2.674, 95%CI −5.185 to −0.164; *p* = 0.037) and lower HOMA-IR (β = −0.731, 95%CI −1.364 to −0.099; *p* = 0.024) levels. No statistically significant interaction between dinner GI and the *FAAH* genotype was detected with respect to glycemia, insulin, and HOMA-IR. Overall, these findings indicate that the dinner GI influences fasting glucose levels in adults with obesity; the *FAAH* variant predicted lower insulin and HOMA-IR levels, supporting a plausible chrono-nutrigenetic interaction between carbohydrate quality, mealtime intake, and *FAAH* variation in metabolic regulation, which must be further studied.

## 1. Introduction

Obesity is a complex and multifactorial disease characterized by excessive accumulation of body fat, which increases the risk of developing cardiometabolic complications such as type 2 diabetes mellitus (T2DM), dyslipidemia, hypertension, and cardiovascular disease (CVD) [1]. Adverse health outcomes result from the interplay between environmental, behavioral, and genetic factors. While lifestyle components, particularly an unbalanced diet and sedentarism, are major drivers of obesity, genetic susceptibility also plays a significant role, both in its onset and progression [2].

Diet plays a critical role in the development and management of obesity and common related complications [2]. Among dietary characteristics, the glycemic index (GI) and glycemic load (GL) are tools used to evaluate the impact of carbohydrate-containing foods on postprandial glycemia [3]. The GI of a food is a measure of how quickly the carbohydrate biomolecules it contains are broken down into glucose during digestion and raise blood glucose levels. Moreover, a meal’s GI reflects the combined effect of multiple compounds present in foods, including its macronutrient composition (carbohydrates, proteins, and lipids) and bioactive compounds such as dietary fibers, polyphenols, and other phytochemicals that modulate glucose absorption and postprandial metabolic responses [4]. Therefore, it is not only the glucose content in food that is important for post-prandial glycemia, as the accompanying biomolecules may also have an impact on glucose homeostasis [5].

Diets with a high GI or GL have been associated with increased insulin resistance and elevated circulating glucose levels, contributing to a higher risk of type 2 diabetes [6]. While the GI and GL are typically assessed based on total daily intake, recent findings highlight that the timing of carbohydrate consumption, or when meals are eaten/mealtimes, particularly in the evening, may significantly influence metabolic responses, including postprandial glucose concentrations, insulin secretion, and subsequent fasting glucose regulation [7,8]. Beyond the quantity and quality of dietary carbohydrates, chrononutrition emphasizes the role of mealtimes in determining metabolic health. Circadian rhythms regulate hormonal secretion, glucose tolerance, and insulin sensitivity, which are generally greater in the morning and decline toward the evening [9]. Accordingly, late consumption of carbohydrate-rich meals, particularly at dinner, has been linked to impaired glucose tolerance, higher postprandial insulin responses, and unfavorable lipid metabolism [8,10]. These findings suggest that not only the glycemic properties of foods but also when they are eaten (mealtime) may critically influence metabolic outcomes among individuals with obesity.

Elevated fasting insulin concentrations and hyperglycemia are linked to inflammatory processes, partly through glucose-induced epigenetic modifications of inflammatory gene promoters and mitochondrial oxidative stress [11]. Evidence from the large multicenter DiOGenes trial demonstrated that low-GI diets were associated with a further decrease in high-sensitivity C-reactive protein (hsCRP) levels after bodyweight loss, independent of diet protein and minor bodyweight variations. These findings suggest that dietary GI not only influences glucose and insulin homeostasis but may also modulate systemic inflammation and cardiovascular risk in subjects with overweight or obesity [12].

In parallel, genetic variants affecting lipid metabolism, appetite regulation, and energy expenditure have been studied in association with obesity and related metabolic disorders [13]. One of these genes is *FAAH* (fatty acid amide hydrolase), which encodes an enzyme involved in the degradation of endocannabinoids [14]. This pathway regulates energy balance, appetite, and glucose metabolism [15]. The single-nucleotide polymorphism rs324420 in the *FAAH* gene produces a missense mutation (C385A, resulting in a Pro129Thr amino acid substitution) that reduces enzymatic activity. Accordingly, individuals may present as CC (Pro/Pro), CA (Pro/Thr), or AA (Thr/Thr). The A allele (Thr129) has been identified as the risk allele and is associated with higher circulating endocannabinoid levels, altered insulin sensitivity, and increased susceptibility to adiposity and metabolic disorders [16]. Furthermore, the *FAAH* gene has been found to be involved in behavior and sex-specific neuroinflammatory alterations induced by life stress in mouse models [17].

Taken together, the interaction between the glycemic properties of dinner-time food consumption, the *FAAH* C385A genetic variant, and markers of insulin metabolism warrants further investigation, particularly in populations with obesity. This is relevant because the GI reflects the combined effect of the biomolecular composition of food or meals and individual glycemic sensitivity [18]. Although *FAAH* is not a core circadian clock gene, it is investigated here as a metabolic modulator that may influence glucose–insulin regulation in response to feeding timing through endocannabinoid signaling. Therefore, this study aims to analyze the association between the dinner GI and the C385A variant in the *FAAH* gene with respect to fasting insulin levels, HOMA-IR, and glucose in adults with obesity. We hypothesized that high-GI evening meals and the *FAAH* C385A variant contribute to increased fasting insulin, HOMA-IR, and glucose concentrations. While previous studies have reported associations between the *FAAH* C385A variant and metabolic traits, this study represents the first analysis of this variant in relation to the dinner GI within a chrononutrition framework.

## 2. Materials and Methods

### 2.1. Study Population

This is a secondary analysis of a cross-sectional study that included 342 Spanish adults, both women and men, with obesity (BMI > 30 kg/m^2^). The study population, recruitment strategy, and general methodology were reported previously using the same cohort [19]. Participants were recruited in Castilla y León, Spain, between 2007 and 2008 through a non-probabilistic sampling method. Primary Care physicians referred patients to the Nutrition Units of the regional Health Areas in Castilla y León for obesity evaluation, during which all assessments described below were conducted (within the study period). Exclusion criteria comprised a personal history of cardiovascular disease or stroke within the past 36 months as well as the use of medications known to influence glucose metabolism or inflammation (e.g., sulfonylureas, metformin, thiazolidinediones, insulin, glucocorticoids, antineoplastic drugs, ACE inhibitors, angiotensin receptor blockers, and psychoactive agents). For this analysis, we included subjects with complete data on glucose and insulin levels and dietary information (*n* = 189). We noticed that some of these participants did not have *FAAH* phenotyping (*n* = 12), but we still maintained them in the descriptive analyses.

To determine the statistical power of the available data, sample size adequacy was evaluated based on the expected frequency of the *FAAH* C385A minor allele in a Spanish sample of diabetic subjects from the same population [20]. Assuming a minor allele frequency of approximately 21%, a significance level of α = 0.05, and a precision of 5%, we estimated that the minimum required sample size was 129 participants. Therefore, the available sample size in this study was considered sufficient for exploratory analyses of genotype–phenotype associations.

The study protocol was approved by the Ethics Committee of Hospital Río Hortega (reference: pi7 542), and written informed consent was obtained from all participants. All procedures complied with the Declaration of Helsinki.

### 2.2. Anthropometric Assessment

Anthropometric measurements were performed according to standardized protocols [21]. Participants wore light clothing and no shoes. Bodyweight was measured with 0.1 kg precision using a calibrated scale (Omron, Los Angeles, CA, USA). Body mass index (BMI) was calculated as body weight (kg) divided by height (m) squared [22].

### 2.3. Biochemical and Clinical Data

Fasting venous blood samples were collected in the morning following at least 8 h of fasting under standardized protocols. Blood samples were collected on days not temporally linked to dietary intake registration, and therefore did not reflect the participants’ consumption on the preceding day. Plasma glucose was measured using an automated glucose oxidase method (Beckman Glucose Analyzer 2, Beckman Instruments, Fullerton, CA, USA), and serum insulin concentrations were determined enzymatically (WAKO Pure-Chemical Industries, Osaka, Japan). Insulin resistance was assessed using the HOMA-IR: [fasting insulin (µU/mL) × fasting glucose (mg/dL)]/405 [23]. No new biological samples were collected or reanalyzed for this study.

### 2.4. Dietary Assessment and Calculation of Glycemic Index and Load

Dietary intake was assessed using two non-consecutive 24 h dietary registers, including one weekend day, without asking about supplements. Registers were reviewed by trained nutritionists to enhance accuracy and included detailed reporting of all food and beverages consumed at each eating occasion. National composition food tables were used as dietary references to estimate total intake of energy, carbohydrates, proteins, lipids, and fiber [24].

Regarding consumption by meal (i.e., eating occasions), the following procedure was carried out: (1) the grams of each food consumed were recorded for each meal (breakfast, morning snack, lunch, afternoon snack, dinner, and evening snack); (2) the average intake of each type of food was calculated based on data from the two 24 h dietary records; and (3) the amounts of fiber, available carbohydrates (CHOs), lipids, proteins, and kilocalories consumed were estimated using the average amount of each food consumed at each meal and the nutrient composition per 100 g of food. All analyses were carried out with a computer-based data evaluation system. The energy and macronutrient composition of each meal were calculated using the BEDCA [25] and CESNID [26] food composition tables.

To obtain the GI, the following formula was used:

GI = Σ (GI_i_ × CHO_i_)/Σ CHO_i_

The GI of each food item (GI_i_) was multiplied by the amount of available carbohydrates in each respective type of food (in grams, CHO_i_) (GI_i_ × CHO_i_). Subsequently, to calculate the GI for each meal, the products corresponding to all foods consumed at each meal were summed separately (Σ (GI_i_ × CHO_i_)). Finally, the sum of these products was divided by the total amount of available carbohydrates in the foods consumed at each meal (Σ CHO_i_). Furthermore, to determine the total daily GI, the products of the GI of each food and its available carbohydrate content were summed across all foods consumed throughout the day, and the result was divided by the total amount of available carbohydrates consumed during the day.

The glycemic load (GL) of each food item was estimated using the following formula: GL_i_ = (GI_i_ × CHO_i_)/100.

Specifically, the GI of each food item (GI_i_) was multiplied by the amount of available carbohydrates (in grams) contained in the respective food (CHO_i_), and the result was divided by 100. The GL of each mealtime was then obtained by summing the GL values of all food items consumed at each meal. The total daily GL was calculated as the sum of the glycemic loads of all individual food items consumed throughout the day.

GI values for individual food items were primarily obtained from the International Tables of Glycemic Index and Glycemic Load Values [27] published by the University of Sydney. When a specific type of food was not available in this database, we consulted the Spanish food composition database BEDCA [25]. If a type of food was not listed there, we subsequently reviewed the CESNID database [26]. For foods not included in any of these sources, the GI was estimated based on the composition of the main ingredients and published reference values for similar foods.

### 2.5. Genotyping

The *FAAH* variant C385A (Pro129Thr) polymorphism was genotyped using real-time PCR with allele-specific fluorescent probes following processes previously reported [28]. Primers and probes were designed with Beacon Designer 4.0 software (Premier Biosoft International, Los Angeles, CA, USA). PCR reactions were conducted in a total volume of 25 µL, containing 50 ng of genomic DNA, 0.5 µL of each primer (forward primer: 5′-ATG TTG CTG GTT ACC CCT CCT C-3′; reverse primer: 5′-CAG GGA CGC CAT AGA GCT G-3′), and 0.25 μL of each probe (wild probe: 5′-FamCTG TCT CAG GCC CCA AGG CAG G-BHQ-1-3′; mutant probe: 5′-Hex-CTG TCT CAG GCC ACA AGG CAG G-BHQ-1-3′). Amplification was performed using an iCycler IQ Thermocycler (Bio-Rad, Hercules, CA, USA) with initial denaturation at 95 °C for 3 min, followed by 50 cycles of 95 °C for 15 s and 59.3 °C for 45 s. Each reaction also included 12.5 µL of IQTM Supermix (Bio-Rad) containing hot-start Taq DNA polymerase.

Allelic discrimination was based on probe hybridization and fluorescence detection: FAM- and HEX-labeled probes specifically recognized the G and A alleles, respectively. Genotypes were determined by measuring the emitted fluorescence during real-time PCR. Hardy–Weinberg equilibrium was assessed to confirm the genetic distribution within the study population.

### 2.6. Statistical Analysis

Continuous variables were presented as means and standard deviations or as medians and 25th and 75th percentiles, depending on the results of the Shapiro–Wilk normality test. Categorical variables were expressed as frequencies and percentages.

Comparisons of quantitative variables between two groups were analyzed using the *t*-test or the Mann–Whitney U test, as appropriate. Comparisons of quantitative variables among three groups were analyzed using ANOVA, while comparisons between qualitative variables were performed using the chi-square test. The Hardy–Weinberg equilibrium for genotype frequencies was also tested using the chi-square test. Due to the low frequency of the AA genotype, CA and AA genotypes were combined and analyzed under a dominant genetic model, with CC homozygotes serving as the reference group.

The correlations between glycemia, insulin, and HOMA-IR and dinner GI, dinner GL, dinner carbohydrates, dinner fiber, dinner proteins, and dinner lipids were analyzed using Spearman’s correlation. The association between glycemia, insulin, and HOMA-IR (as dependent variables) and dinner GI, total GI, and the *FAAH* variant was analyzed using linear regression adjusted for sex, age, BMI, energy intake, and dinner protein. The beta value (β), with its 95% confidence interval (95%CI) and *p*-value, is shown.

Statistical analyses were conducted using IBM SPSS Statistics (version 20; IBM Corp., Armonk, NY, USA). Statistical significance was set at *p* < 0.05. The graphics were made with RStudio (version 2024.12.1+563; Posit Software, PBC) and Microsoft Excel (version 365; Microsoft Corp., Redmond, WA, USA).

## 3. Results

### 3.1. Sample Characteristics

A total of 189 Spanish adults (129 women) with obesity were included in the analysis (mean age, 41 ± 12 years; mean BMI, 38.0 ± 5.2 kg/m^2^). Sex differences were observed in regard to dietary intake and biochemical markers: men had higher energy intake, total daily GI values, total daily GL, fasting insulin levels, and HOMA-IR levels than women. Subjects aged 41 years or younger had higher total daily GI values and lower glycemia levels than older participants. Participants with a BMI above the median (>37.30 kg/m^2^) had higher fasting glycemia, insulin, and HOMA-IR levels. The distribution of the *FAAH* C385A variant did not differ by sex, age, or BMI (Table 1). Likewise, no significant differences were observed in regard to insulin, glycemia, HOMA-IR levels, or energy and macronutrient intake throughout the day or at dinner, according to the allele distribution. However, a significant difference in the percentage of carbohydrates consumed during the day was observed: subjects with the C385C variant had a higher percentage than those with the C385A + A385A variant. Similarly, a trend towards higher insulin and HOMA-IR levels was observed in subjects with the C385C variant (Supplementary Table S1).

### 3.2. Dietary Characteristics Stratified by Metabolic Categories

When the participants were stratified by fasting glycemia, insulin, and HOMA-IR (Table 2) levels, those with lower fasting glucose levels had higher total GI and dinner GI values than those with higher fasting glucose levels. Groups with higher insulin and HOMA-IR levels had diets with higher total daily GI values. Among participants with higher insulin levels, total fiber consumption was lower, and protein consumption at dinner was higher. In terms of HOMA categories, subjects with higher HOMA-IR values showed significantly greater protein intake at dinner. Across tertiles of glycemia, total daily GI and dinner GI differed significantly (Supplementary Table S2). Across insulin and HOMA-IR tertiles, total daily GI and lunch GI also differed significantly.

### 3.3. Associations Between Dinner GI and Glycemia, Insulin, and HOMA-IR Levels

A significant correlation was observed between dinner GI, glycemia (rho: −0.253, *p* < 0.001), and insulin (rho: 0.156, *p* = 0.032) (Supplementary Figure S1).

In linear regression models (Table 3, Supplementary Table S3), dinner GI was inversely associated with fasting glycemia after adjustment (β: −0.172; 95%CI: −0.298, −0.045), whereas its association with insulin (β: 0.084; 95%CI: −0.009, 0.177) was attenuated after adjustment for the covariates sex, age, BMI, energy intake, *FAAH* variant, and dinner protein (included because of the significant correlation with insulin and HOMA-IR). No significant association was observed between dinner GI and HOMA-IR.

In contrast, total daily GI was consistently associated with higher fasting insulin (β: 0.302; 95%CI: 0.127, 0.477) and HOMA-IR (β: 0.056; 95%CI: 0.012, 0.100) levels, and no-significantly with lower fasting glycemia levels (β: −0.240; 95%CI: −0.484, 0.004), in fully adjusted models (Supplementary Table S3).

In multivariable regression, we observed that sex and BMI were independent predictors of fasting glycemia, insulin, and HOMA-IR levels. Age was an independent predictor of insulin and glycemia levels. *FAAH* genotype was independently associated with fasting insulin and HOMA-IR levels (Table 3).

**Figure 1:**
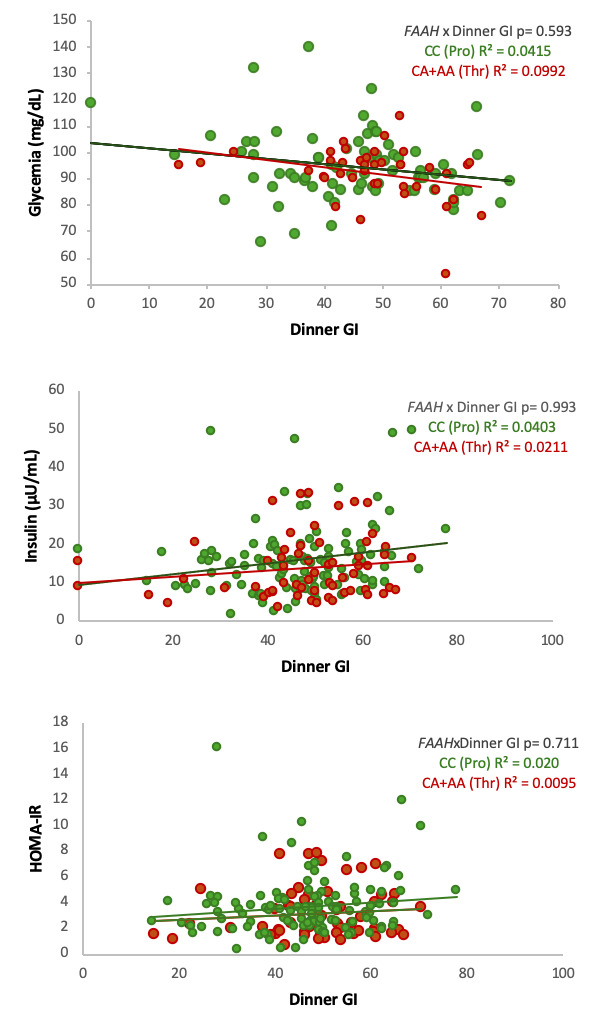
Association between the dinner glycemic index (GI) and glycemia (**first panel**), insulin (**second panel**), and HOMA-IR (**third panel**) levels according to *FAAH* C385A genetic variant. Scatter plots depict the relationship between dinner GI and fasting glycemia, fasting insulin, and HOMA-IR levels, respectively. Green circles represent individuals with the CC genotype (Pro129Pro), while red circles represent carriers of the A allele (CA: Pro129Thr and AA: Thr129Thr). Solid lines indicate linear regression models for each genotype group. The *p* value shown corresponds to the interaction term between dinner GI and *FAAH* genotype in the adjusted regression models.

No significant interaction between dinner GI and *FAAH* genotype was observed for fasting glucose, insulin, or HOMA-IR levels (Figure 1).

## 4. Discussion

This study provides novel evidence linking chrono-nutrition, dietary GI (as a mixture of biomolecules), and genetic variation in *FAAH* with fasting metabolic outcomes among adults with obesity. The main finding was that dinner GI showed a consistent inverse association with fasting glycemia, even after adjustment for age, sex, BMI, energy intake, *FAAH* risk allele, and dinner protein. In this context, the GI concept was designed to account for not only glucose intake but also the putative impact of associated macromolecules, micronutrients, and nutritional biofactors on glycemia [29]. Additionally, total daily GI was positively associated with fasting insulin concentrations and HOMA-IR levels while also showing an inverse relationship with fasting glycemia. Furthermore, the *FAAH* C385A variant was independently associated with fasting insulin and HOMA-IR levels. Together, these results suggest that both the quality and timing of carbohydrate intake, in combination with genetic predisposition, contribute to fasting metabolic profiles in individuals with obesity.

Previous large-scale evidence has consistently shown the detrimental metabolic impact of high-GI and high-GL diets. In three major United States cohorts encompassing more than 3.8 million person-years of follow-up and over 15,000 incident cases of patients with type 2 diabetes, Bhupathiraju et al. [6] reported that the participants in the highest GI quintile had a 33% greater risk of developing type 2 diabetes, while those in the highest GL quintile had a 10% higher risk, particularly when combined with low cereal fiber intake. Recent work incorporating chrono-nutrition suggests that carbohydrate timing intake modifies glycemic responses. Circadian regulation leads to greater insulin sensitivity and glucose tolerance in the morning, with a progressive physiological decline toward the evening, as highlighted by Henry et al. [30], supporting the concept that misaligned eating patterns may impair glycemic responses.

Evidence from time-restricted eating studies supports the importance of meal timing in metabolic regulation. In a randomized crossover trial, restricting the daily eating window to earlier hours improved glucose tolerance, fasting insulin levels, and insulin sensitivity compared with late eating, independent of energy intake. These findings suggest that aligning food intake with circadian rhythms may enhance metabolic control and reinforce the notion that eating later in the day can impair glycemic responses [31]. Similarly, in a crossover study, Morgan et al. [8] demonstrated that consuming high-GI meals late in the evening resulted in higher interstitial glucose levels and impaired postprandial insulin sensitivity compared to equivalent meals consumed earlier in the day. In a clinical weight loss trial in which dinner carbohydrate intake was increased, higher levels of glycemic biomarkers were observed; however, the magnitude of the increase differed when the high-CHO meal was consumed at lunch [32]. These findings support the concept that late high-GI meals exacerbate circadian misalignment of glucose metabolism, probably involving regulatory mechanisms like decreasing insulin sensitivity [33] associated with the concurrence of food intake and high levels of melatonin [34], and may contribute to metabolic dysregulation, which is also affected by genetic factors [35]. Also, chronotype is a biologically plausible modifier of the relationship between meal timing, carbohydrate quality, and metabolic regulation [36]. Future studies should explicitly address circadian misalignment between endogenous metabolic rhythms and behavioral patterns, such as meal timing and sleep–wake cycles.

Complementary evidence from bodyweight-loss maintenance studies also highlights the role of dietary GI. The DiOGenes multicenter trial showed that diets with modestly higher protein content or lower GI values improved long-term bodyweight maintenance following an initial weight reduction. Participants in the high-protein or low-GI groups regained less body weight compared with those adhering to low-protein or high-GI diets, demonstrating that modest dietary adjustments can sustain bodyweight loss and improve adherence [37]. Moreover, the timing of caloric distribution has been directly compared in intervention studies. In a 12-week randomized trial conducted on overweight and obese women with metabolic syndrome, Jakubowicz et al. [10] reported that a high-calorie breakfast led to greater reductions in bodyweight and waist circumference than an isocaloric diet involving high caloric intake at dinner. The breakfast-rich diet also yielded more pronounced improvements in fasting glucose, insulin, HOMA-IR, lipid profile, and postprandial responses, underscoring the metabolic advantages of concentrating caloric and carbohydrate intake earlier in the day.

In this study, the positive associations between dietary GI, insulin, and HOMA-IR levels observed are consistent with prior reports [38,39]. Liese et al. [39], in the Insulin Resistance Atherosclerosis Study, observed that neither GI nor GL remained independently associated with insulin sensitivity after multivariate adjustments. However, significant associations between GL and total carbohydrate intake were present before adjusting for energy intake, suggesting that the glycemic characteristics of the diet may contribute to early metabolic disturbances prior to the influence of overall caloric consumption. Similarly, O’Sullivan et al. [38] found that GL, particularly at lunch, independently predicted higher HOMA-IR levels in older women. These results support the notion that diets with a higher glycemic impact can contribute to early alterations in glucose–insulin homeostasis, a finding consistent with the pattern observed in our cohort.

Taken together, previous studies generally indicate that late high-GI meals impair glucose handling. In this context, the inverse association between dinner GI and fasting glucose observed in our study could still be compatible with compensatory increases in insulin secretion to preserve euglycemia in early stages of insulin resistance [40]. This stage is clinically relevant because it remains amenable to improvement through lifestyle and pharmacological interventions. For example, Azócar-Gallardo et al. [41] demonstrated that concurrent training, with or without metformin, improved HOMA-IR and fasting insulin levels in individuals with early insulin resistance.

At the molecular level, circadian regulation of glucose–insulin homeostasis is mediated not only by systemic hormonal rhythms but also by peripheral metabolic clocks [30]. Core clock proteins, particularly CLOCK and BMAL1, regulate the transcription of genes involved in β-cell function, hepatic glucose production, and insulin signaling. An experimental disruption of CLOCK or BMAL1 in animal models results in reduced insulin secretion, impaired glucose tolerance, and increased adiposity. SIRT1, a nutrient- and redox-sensitive deacetylase, directly modulates clock activity by promoting BMAL1–CLOCK transcriptional function and has been shown to link metabolic state with circadian gene expression [42]. Reduced SIRT1 activity or circadian misalignment leads to decreased insulin sensitivity and increased oxidative and metabolic stress [43]. In this context, late carbohydrate intake, occurring during a phase of reduced circadian metabolic efficiency, may influence fasting glucose regulation, especially in subjects with the rs3749474 variant of the *CLOCK* gene [44], a finding that is consistent with the inverse association observed in this study between dinner GI and fasting glycemia.

The endocannabinoid system is another important modulator of energy balance and glucose metabolism [45]. FAAH, the main enzyme responsible for degrading endocannabinoids such as anandamide (AEA), influences insulin sensitivity and lipid storage [46]. The *FAAH* C385A polymorphism (Pro129Thr) reduces FAAH activity and increases levels of circulating endocannabinoids, effects that have been associated with obesity, glucose–insulin homeostasis, and cardiometabolic risk [47,48]. Interestingly, in our study, the A allele was associated with lower fasting insulin and HOMA-IR levels after adjustment. This may reflect differences in genetic background, environmental exposure, disease stage, or dietary patterns. Recent reviews have highlighted that associations between the *FAAH* C385A variant and metabolic traits are inconsistent across studies and populations, underscoring the context-dependent expression of this polymorphism [49,50].

From a molecular perspective, the metabolic effects associated with the *FAAH* C385A variant can be interpreted through endocannabinoid signaling. Reduced FAAH activity results in higher circulating anandamide levels, which increases activation of the cannabinoid receptor CB1 to promote orexigenic drive, lipogenesis, and mitochondrial oxidative capacity [51]. In several reports, this phenotype correlates with increased adiposity and insulin dysregulation [49]. However, its metabolic impact is not uniform. An increasing amount of evidence is showing that obesity is not metabolically homogeneous. Individuals with comparable adiposity may differ markedly in terms of glucose tolerance, fasting insulin levels, systemic inflammation, and cardiometabolic risk, giving rise to metabolically healthy and unhealthy obesity phenotypes [52]. Such variability may result from differences in adipose tissue function, hormonal adaptation, and genetics [53]. Within this framework, the lower fasting insulin and HOMA-IR levels observed in A-allele carriers may reflect a compensated metabolic profile despite excess weight.

Other biological systems may contribute to the variability in insulin resistance. For instance, Aljuraiban et al. [54] showed that gut microbiome composition was associated with fasting insulin levels, HOMA-IR levels, and adiposity distribution in women with obesity, reinforcing the multifactorial nature of metabolic dysregulation.

The interaction between dietary factors and genetic variation is a central topic in nutrigenetics. No significant interaction was detected in this study; however, our data indicate a plausible dinner-specific GI–*FAAH*–glucose/insulin regulatory nexus. This chrono-nutrigenetic perspective suggests that dietary carbohydrate quality and timing may differentially affect individuals according to their genetic background. While most previous nutrigenetic studies focused on macronutrient proportions or total GI/GL [55], our data emphasize the importance of evaluating the timing of carbohydrate intake. Future intervention studies should test whether evening low-GI meals confer metabolic advantages in individuals carrying the *FAAH* A allele.

These findings support the need to move beyond total daily nutrient intake and incorporate both timing of intake and genetic susceptibility into dietary recommendations. Personalized nutrition approaches could benefit individuals with obesity by tailoring carbohydrate quality not only by quantity and type but also by the timing of consumption. In clinical practice, counseling patients with obesity to reduce dinner GI values may represent a simple strategy for improving fasting glucose–insulin balance, particularly among those genetically predisposed to issues. Researchers should study whether the time at which foods with a high GI value or meals with a high GL are consumed is also associated, in longitudinal studies, with a higher risk of developing type 2 diabetes [56] and whether carrying this variant could involve a greater disease burden.

This study has several notable strengths. A major strength is its comprehensive characterization of the participants (selected with clearly defined inclusion and exclusion criteria), integrating anthropometric, biochemical, dietary, and genetic data (variables obtained with standardized protocols and by trained personnel), enabling a multifactorial evaluation of chrononutrition, carbohydrate quality, and *FAAH C385A* genetic variability. Its precise estimation of GI at each eating occasion, including a specific calculation for dinner GI, represents an important methodological advantage over previous studies, which focused solely on total daily GI/GL. The use of multivariable models adjusted for relevant confounders, such as age, sex, BMI, energy intake, *FAAH* genotype, and dinner protein, further reinforces the internal validity of the findings. In any case, the role of dietary protein in our findings may require further attention. For example, dietary protein, in conjunction with glycemic index, may affect metabolic outcomes and should not be discarded in future studies.

Nevertheless, some limitations must be acknowledged. This study’s cross-sectional design precludes causal inference between dinner GI, FAAH genotype, and fasting glucose–insulin outcomes. This study was conducted exclusively among adults with obesity, a population characterized by altered glucose–insulin regulation. Therefore, these results cannot be generalized to non-obese or metabolically healthy populations. Future studies conducted across diverse metabolic phenotypes are needed. Dietary assessment based on self-reported 24 h records may introduce recall bias and poses a risk of underreporting, particularly for individuals with obesity. Only a single genetic variant within the endocannabinoid pathway was examined, without considering additional genes or molecular markers (e.g., inflammatory or oxidative stress parameters) that could have provided deeper mechanistic insights [17]. Moreover, although the sample size was sufficient to detect the main genetic and dietary associations, identification of subtle gene–diet interaction effects may require larger samples. Additionally, the lack of circadian phase markers and sleep parameters limited our ability to directly assess circadian misalignment or postprandial metabolic responses and therefore warrants a cautious interpretation of the chrononutrigenetic implications of *FAAH*. Despite these limitations, this study makes a novel contribution by integrating chrononutritional and nutrigenetic perspectives to help us to better understand metabolic regulation in adults with obesity, although the data were collected several years ago. However, the novelty of this work lies not in the timing of data collection but in the research question, conceptual framework, and interpretation, which are both innovative and timely. We consider reexamining well-characterized datasets through emerging scientific perspectives —such as chrononutrition and nutrigenetics—to be a valid and valuable approach that can make a meaningful contribution to the current literature.

Notably, the GI represents a combination of biomolecules that influence post-prandial glycemia in addition to glucose intake [4,57,58]. We connect our findings with these factors by examining how dietary patterns and genetic variants may interact with internal circadian regulation to shape metabolic risk profiles, particularly because the *FAAH* gene is involved in behavior and sex-specific neuroinflammatory alterations induced by life stress [17].

## 5. Conclusions

This study provides new evidence integrating chrononutritional and nutrigenetic perspectives to better explain glucose–insulin regulation in obesity. Our findings indicate that both the glycemic quality and the timing of carbohydrate intake are relevant determinants of fasting metabolic balance and that genetic variation in the *FAAH* gene may further modulate these responses. Together, these results suggest that metabolic regulation is influenced not only by what individuals eat but also by when they eat, along with genetic background. Incorporating dietary composition, meal timing, and genetic susceptibility into dietary strategies should be considered an emerging component of precision nutrition rather than a standard clinical approach, in part due to the current costs associated with genetic testing. This study represents an initial step toward identifying biologically meaningful subgroups that may benefit from tailored dietary strategies. Our findings should be interpreted as hypothesis-generating; therefore, further longitudinal and clinical trials conducted using larger samples would help confirm these associations and explore whether low-glycemic-index evening meals may improve fasting and postprandial metabolic outcomes, particularly when stratified by genetic variants involved in glucose and lipid metabolism, such as *FAAH*. Although widespread implementation is not yet feasible, advances in genotyping technologies and the accumulation of evidence may, in the future, enable the integration of such approaches into clinical and public health practice.

## Figures and Tables

**Table 1:** Nutritional, biochemical, and genetic characteristics in adults with obesity according to sex, age, and BMI categories.

	Sex			Age			BMI		
	Men *n* = 60	Women *n* = 129		≤41 years *n* = 94	>41 years *n* = 95		≤37.30 kg/m^2^ *n* = 95	>37.30 kg/m^2^ *n* = 94	
	Mean ± SD	Mean ± SD	*p*	Mean ± SD	Mean ± SD	*p*	Mean ± SD	Mean ± SD	*p*
Women *n (%)*	NA	129 (68.3)	NA	65 (69.1)	64 (67.4)	0.793	60 (63.2)	69 (73.4)	0.130
Age (years)	41 ± 9	41 ± 13	0.653	31 ± 6	51 ± 7	<0.001	40 ± 12	42 ± 12	0.117
BMI (kg/m^2^)	37.3 ± 5.4	38.2 ± 5.1	0.266	37.5 ± 5.2	38.3 ± 5.2	0.289	33.7 ± 2.0	42.2 ± 3.8	<0.001
Glycemia (mg/dL) *	95.2 ± 13.6	93.1 ± 11.4	0.367	89.2 ± 9.7	98.3 ± 12.7	<0.001	90.7 ± 11.9	96.9 ± 11.7	<0.001
Energy intake (Kcal) *	2366.8 ± 972.6	1890.2 ± 655.5	<0.001	2068 ± 832.2	2015.3 ± 768.9	0.719	2077.7 ± 782.3	2005 ± 818.7	0.390
Total daily GI	53.2 ± 7.6	50.7 ± 6.7	0.023	52.6 ± 7	50.4 ± 7.1	0.034	51 ± 6.7	52 ± 7.5	0.334
Total daily GL *	96.2 ± 44.1	80.1 ± 39.1	0.011	87.8 ± 43.1	82.7 ± 39.5	0.492	83.1 ± 38.3	87.3 ± 44.2	0.826
HOMA-IR *	4.3 ± 3.0	3.4 ± 1.9	0.032	3.7 ± 2.5	3.7 ± 2.1	0.936	3.1 ± 1.9	4.2 ± 2.6	<0.001
Insulin (μU/mL) *	18.2 ± 11.5	14.5 ± 7.7	0.040	16.5 ± 9.9	14.9 ± 8.5	0.293	13.8 ± 8.2	17.6 ± 9.9	0.002
	*n* (%)	*n* (%)	*p*	*n* (%)	*n* (%)	*p*	*n* (%)	*n* (%)	*p*
*FAAH* *C385A*			0.244			0.748			0.597
CC	32 (59.3)	84 (68.3)		60 (66.7)	56 (64.4)		60 (67.4)	56 (63.6)	
CA + AA	22 (40.7)	39 (31.7)		30 (33.3)	31 (35.6)		29 (32.6)	32 (36.4)	

BMI: body mass index; NA: not applicable; GI: glycemic index; GL: glycemic load; FAAH: fatty acid amide hydrolase. Age and BMI were divided according to the 50th percentile. One hundred seventy-seven participants had genetic data available. A dominant model was applied, comparing carriers of the A allele (CA + AA) with wild-type CC individuals. Quantitative variables are expressed as means and standard deviations, and qualitative variables are expressed as frequencies and percentages. * The data are shown in the original units, but the statistical analyses were performed using log transformed values. The difference in quantitative variables was analyzed using a *t*-test. The difference between qualitative variables was analyzed using the Chi^2^ test. *p* < 0.05 was considered significant.

**Table 2:** Diet consumed throughout the day and at dinner among adults with obesity according to glycemia, insulin, and HOMA-IR categories.

	Glycemia			Insulin			HOMA-IR		
	≤93.0 mg/dL *n* = 98	>93.0 mg/dL n = 91		≤14.40 μU/mL *n* = 95	>14.40 μU/mL *n* = 94		≤3.27 *n* = 95	>3.28 *n* = 94	
	Median (P25, P75)	Median (P25 y P75)	*p*	Median (P25, P75)	Median (P25, P75)	*p*	Median (P25 y P75)	Median (P25, P75)	*p*
Total Energy intake (Kcal)	1951.7 (1553.6, 2344.1)	1886.7 (1471.8, 2285.6)	0.371	1828 (1449.4, 2246.3)	2029.5 (1562.9, 2344.1)	0.224	1849.4 (1450.7, 2285.6)	2015.8 (1556.3, 2344.1)	0.406
Total CHO (g)	199.5 (141.8, 247.8)	177.5 (126, 236)	0.126	178.5 (134.6, 243.6)	195 (143, 241.2)	0.209	185.3 (136.9, 243.6)	192.3 (143.0, 241.2)	0.444
Total % CHO	40.5 (33.0, 46.0)	41.0 (32, 45)	0.839	40 (33, 44)	41.0 (32.0, 46.0)	0.486	40.0 (33.0, 44.0)	41.5 (32.0, 46.0)	0.258
Total Fiber (g)	15 (9.9, 20.9)	15.4 (11.8, 19.1)	0.697	16.3 (12.1, 22.5)	13.8 (9.0, 18.7)	0.012	16.0 (11.7, 21.6)	13.9 (9.1, 19.0)	0.062
Total GI	52.6 (47.4, 58.1)	49.7 (45.8, 55.1)	0.008	49.1 (45.0, 54.8)	52.6 (48.1, 57.9)	0.001	49.9 (45.0, 54.8)	52.5 (47.9, 57.5)	0.008
Total GL	87.7 (58.9, 111.2)	76.8 (51.1, 99.5)	0.069	75.9 (52, 99.1)	90.6 (58.9, 111.0)	0.071	76.0 (51.7, 103.4)	89.9 (59.7, 110.5)	0.083
Total PP (g)	88.9 (75.9, 103.8)	88.0 (73.5, 105.8)	0.488	86.3 (73.5, 98.3)	91.8 (74.4, 107.1)	0.176	88.0 (73.5, 99.4)	90.0 (74.4, 107.1)	0.244
Total % of PP	18.0 (16.0, 22.0)	19.0 (18, 22)	0.155	19.0 (17.0, 22)	19.0 (16.0, 23.0)	0.898	19.0 (17.0, 21.0)	19.0 (16.0, 23.0)	0.537
Total LP (g)	85.2 (66.9, 108.6)	79.7 (60.5, 103.5)	0.272	80.7 (62, 107.8)	84.1 (66.9, 103.5)	0.603	85.7 (62.8, 107.8)	80.9 (65.5, 103.5)	0.970
Total % of LP	40.0 (35.0, 47.0)	41 (34, 47)	0.840	41.0 (35.0, 48.0)	40.0 (35.0, 45.0)	0.311	41.0 (36.0, 48.0)	40.0 (34.0, 45.0)	0.227
Dinner Energy Intake (Kcal)	493.1 (322.1, 680.3)	457.6 (322.1, 601.5)	0.445	442.6 (321.5, 579.3)	516 (328.1, 657.3)	0.215	449.3 (322.1, 594.6)	509 (325.3, 657.3)	0.404
Dinner CHO (g)	33.1 (19.2, 48.9)	31.2 (14, 46.1)	0.176	34 (17.5, 44.3)	31.5 (16.9, 50.2)	0.780	34.0 (17.5, 44.3)	31.5 (17.3, 49.8)	0.722
Dinner Fiber (g)	3.5 (2.0, 5.6)	3.9 (1.7, 6.4)	0.739	4.1 (2.3, 6.4)	2.8 (1.7, 6.0)	0.060	4.0 (2.2, 6.5)	2.9 (1.7, 6.0)	0.131
Dinner GI	49.3 (41.6, 59.7)	47.1 (39.2, 51.0)	0.014	47.5 (39.1, 55.4)	48.4 (41.2, 57.1)	0.356	47.8 (39.1, 56.0)	48.3 (41.2, 55.6)	0.546
Dinner GL	17 (8.5, 25.4)	14.3 (6.4, 21.5)	0.071	15.9 (8.0, 23.3)	15.0 (7.4, 24.7)	0.728	16.6 (8.0, 23.3)	15.0 (7.9, 24.6)	0.734
Dinner PP (g)	26.7 (19.5, 38.5)	27.4 (17.7, 38.5)	0.867	25.0 (17.5, 36.2)	29.0 (20.7, 41.0)	0.048	24.7 (17.7, 36.2)	29.1 (20.7, 41.0)	0.046
Dinner LP (g)	24.5 (16, 33.4)	23.4 (16.9, 30.2)	0.545	22.5 (16.6, 30.8)	25.4 (16.1, 33.7)	0.431	23.9 (16.6, 32.2)	24.0 (16.1, 32.3)	0.985

P: percentile, CHOs: carbohydrates; GI: glycemic index; GL: glycemic load, LP: lipids; PP: proteins. Glycemia, insulin, and HOMA-IR were divided according to the 50th percentile. Variables are expressed as medians and 25th and 75th percentiles. The difference in medians was calculated using the Mann–Whitney U test. Statistical significance was set at *p* < 0.05.

**Table 3:** Multivariate analysis of nutritional, biochemical, and genetic parameters associated with glycemia, insulin, and HOMA-IR levels in adult subjects with obesity.

Glycemia (mg/dL)	β (95%CI)	*p*
Sex	−4.359 (−8.100, −0.618)	0.023
Age (years)	0.339 (0.203, 0.475)	<0.001
BMI (Kg/m^2^)	0.343 (0.036, 0.650)	0.029
Energy intake (Kcal)	−0.0001 (−0.002, 0.002)	0.912
Dinner GI	−0.172 (−0.298, −0.045)	0.008
*FAAH* C385A	−3.062 (−6.481, 0.358)	0.079
Dinner Protein (g)	−0.019 (−0.112, 0.073)	0.679
Final R^2^		0.225
**Insulin (μU/mL)**	**β (95%CI)**	***p***
Sex	−3.483 (−6.230, −0.737)	0.013
Age (years)	−0.095 (−0.195, 0.004)	0.061
BMI (Kg/m^2^)	0.533 (0.308, 0.759)	<0.001
Energy intake (Kcal)	−0.001 (−0.003, 0.001)	0.276
Dinner GI	0.084 (−0.009, 0.177)	0.077
*FAAH* C385A	−2.674 (−5.185, −0.164)	0.037
Dinner Protein (g)	0.059 (−0.009, 0.127)	0.087
Final R^2^		0.204
**HOMA-IR**	**β (95%CI)**	***p***
Sex	−1.005 (−1.696, −0.313)	0.005
Age (years)	−0.009 (−0.034, 0.016)	0.463
BMI (Kg/m^2^)	0.137 (0.080, 0.194)	<0.001
Energy intake (Kcal)	−0.0002 (−0.0006, 0.0002)	0.348
Dinner GI	0.008 (−0.015, 0.032)	0.491
*FAAH* C385A	−0.731 (−1.364, −0.099)	0.024
Dinner Protein (g)	0.014 (−0.003, 0.031)	0.105
Final R^2^		0.174

BMI: body mass index, GI: glycemic index, *FAAH*: fatty acid amide hydrolase gene. Glycemia and insulin levels correspond to fasting measurements obtained after an overnight fast; HOMA-IR was calculated using the formula: [fasting insulin (µU/mL) × fasting glucose (mg/dL)]/405.

## Data Availability

The data that support the findings of this study are available on request from the corresponding author due to use of health data that are highly protected through the current regulations in our country.

## Supplementary Materials

The following supporting information can be downloaded at https://www.mdpi.com/article/10.3390/biom16020274/s1. Figure S1: Heat map of Spearman’s Rho related to different dietary and biochemical variables. Table S1: Characteristics of participants according to FAAH C385A variant. Table S2: Glycemic indexes by eating occasion, according to tertiles of fasting glycemia, insulin, and HOMA-IR. Table S3: Associations of dinner glycemic index and total daily glycemic index with insulin, fasting glycemia, and HOMA-IR, in adults with obesity.

## Abbreviations

The following abbreviations are used in this manuscript:

ANOVA	Analysis of variance
BMI	Body mass index
CB1	Cannabinoid receptor 1
CHO	Carbohydrates
CVD	Cardiovascular disease
DNA	Deoxyribonucleic acid
FAAH	Fatty acid amide hydrolase
GI	Glycemic index
GL	Glycemic load
HOMA-IR	Homeostatic model assessment for insulin resistance
hsCRP	High-sensitivity C-reactive protein
LP	Lipids
NA	Not applicable
P	Percentile
PCR	Polymerase chain reaction
PP	Proteins
T2DM	Type 2 diabetes mellitus
