# Supplementary material for: Plausible Obesity-Related Chronometabolic and Nutrigenetic Nexus Concerning Dinner Glycemic Index and the FAAH C385A Variant
Source: Biomolecules. 2026 Feb 9;16(2):274. doi: 10.3390/biom16020274 (PMC12937749; doi:10.3390/biom16020274)
Supplement: Supplementary file 1 [file biomolecules-16-00274-s001.zip › biomolecules-4046740-supplementary.pdf]

Supplementary Materials

Table S1. Characteristics of participants according to *FAAH* C385A variant using a dominant model, comparing carriers of the A allele (CA+AA) to wild-type CC individuals.

	FAAH C385A genotypes		
	CC	CA + AA	
	Mean ± SD	Mean ± SD	p
Age (years)	41 ± 12	42 ± 13	0.599
BMI (kg/m^2)	37.8 ± 5.4	38.2 ± 5.1	0.594
Insulin (µU/mL)*	15.9 ± 9.1	13.9 ± 7.9	0.099
Glycemia (mg/dL)*	94.3 ± 13.0	91.8 ± 10.0	0.235
HOMA-IR*	3.7 ± 2.3	3.2 ± 1.9	0.068
	Median (P25 y P75)	Median (P25, P75)	p
Total Energy intake (Kcal)	1876.6 (1506.7, 2251.2)	2066.1 (1630.0, 2353.6)	0.371
Total CHO (g)	189.6 (148.1, 244.4)	190.3 (120.7, 236.0)	0.595
Total percentage of CHO (%)	42.0 (34.5, 46.0)	38.0 (31.0, 43.0)	**0.025**
Total fiber (g)	15.5 (11.3, 20.6)	15.4 (10.6, 19.1)	0.600
Total GI	50.9 (46.6, 56.8)	50.5 (46.6, 55.4)	0.606
Total GL	81.3 (59.8, 110.7)	81.5 (54.7, 103.9)	0.389
Total PP (g)	87.2 (73.7, 103.3)	90.7 (75.9, 115.9)	0.279
Total percentage of PP (%)	18.5 (16.0, 22.0)	19.0 (17.0, 22.0)	0.536
Total LP (g)	81.3 (61.8, 102.5)	85.2 (66.9, 107.6)	0.369
Total percentage of LP (%)	40.0 (35.0, 46.0)	41.0 (35.0, 48.0)	0.347
Dinner Energy intake (Kcal)	461.4 (323.0, 631.2)	489.3 (344.3, 680.0)	0.533
Dinner CHO (g)	32.1 (17, 47.4)	37.3 (21.4, 49.9)	0.322
Dinner fiber (g)	3.4 (1.9, 6.3)	3.9 (2.5, 6.0)	0.637
Dinner GI	47.5 (38.7, 55.2)	49.7 (42.9, 58.0)	0.178
Dinner GL	15.1 (7.3, 23.3)	17.3 (10.9, 25.1)	0.273
Dinner PP (g)	26 (18.9, 36.8)	28.8 (18.8, 41.4)	0.316
Dinner LP (g)	23.8 (15.9, 33.5)	25.1 (17.9, 31.9)	0.686

FAAH: fatty acid amide hydrolase, CHO: carbohydrates; GI: glycemic index; GL: glycemic load; LP: lipids; PP: proteins.

*The data are shown in the original units, but the statistical analyses were performed with their log transformation.

One hundred seventy-seven participants had genetic data available.

Quantitative variables are expressed as means and standard deviations, or as medians and the 25th and 75th percentiles. The difference in quantitative variables was analyzed using a t-test or a Mann-Whitney U test, as appropriate. Statistical significance was set at $p<0.05$

Table S2. Glycemic indexes by eating occasion, according to tertiles of fasting glycemia, insulin, and HOMA-IR.

	Glycemia				Insulin				HOMA-IR			
	≤ 88.0 mg/dL	>88.0 - ≤98.0 mg/dL	> 98.0 mg/dL		≤ 10.5 µU/mL	>10.5 – ≤16.76 µU/mL	> 16.76 µU/mL		≤ 2.38	>2.39 – ≤3.95	> 3.96	
	n= 63	n= 67	n= 59		n= 63	n= 63	n= 63		n= 63	n= 62	n= 64	
	Mean ± SD	Mean ± SD	Mean ± SD	p	Mean ± SD	Mean ± SD	Mean ± SD	p	Mean ± SD	Mean ± SD	Mean ± SD	p
Breakfast GI	48.5 ± 10.8	47.8 ± 9.5	48.2 ± 9.2	0.930	46.4 ± 7.1	49.2 ± 9.9	48.9 ± 11.8	0.221	46.6 ± 7.6	49.0 ± 9.9	48.8 ± 11.5	0.321
Morning snack GI	44.4 ± 20.8	48.6 ± 18.2	45.7 ± 17.5	0.579	43.7 ± 14.1	45.9± 24.5	49.2 ± 15.3	0.415	42.7 ± 15.0	48.9 ± 23.6	47.2 ± 16.5	0.300
Lunch GI	54.9 ± 11.0	52.0 ± 9.7	52.2 ± 11.2	0.248	49.8 ± 11.0^{a}	56 ± 8^{b}	53.2 ± 11.8^{ab}	**0.005**	49.6 ± 10.9^{a}	56.6 ± 7.9^{b}	52.9 ± 11.6^{ab}	**0.001**
Afternoon snack GI	49.2 ± 16.2	45.2 ± 16.8	47.9 ± 21.7	0.502	46.4 ± 15	45.7 ± 19.8	50.4 ± 19.4	0.387	45.2 ± 15.9	49.3 ± 17.3	47.3 ± 21.5	0.514
Dinner GI	51.2 ± 11.8^{a}	47.3 ± 12.2^{ab}	42.0 ± 14.6^{b}	**0.001**	44.2 ± 13.9	46.9 ± 12.2	49.6 ± 13.5	0.078	44.5 ± 13.8	47.5 ± 11.3	48.7 ± 14.5	0.195
Late-night snack GI	51.7 ± 25.3	40.7 ± 21.9	42.7 ± 14.6	0.305	42.0 ± 19.0	48.3 ± 25.2	43.2 ± 17.9	0.653	46.8 ± 25.6	42.8 ± 16.2	43.0 ± 19.4	0.809
Total daily GI	53.3 ± 6.1^{a}	50.5 ± 7.3^{a}	50.5 ± 7.6^{a}	**0.030**	48.5 ± 6.4^{a}	52.8 ± 6.8^{b}	53.2 ± 7.2^{b}	**<0.001**	48.5 ± 6.4^{a}	53.1 ± 6.6^{b}	52.9 ± 7.4^{b}	**<0.001**

GI: glycemic index. Glycemia, insulin, and HOMA-IR were divided into tertiles. Variables are expressed as mean and standard deviation. The difference in quantitative variables was analyzed using ANOVA. A post hoc analysis with Bonferroni correction was performed on significant results. Different letters indicate significant differences. Statistical significance was set at $p<0.05$.

Table S3. Associations of dinner glycemic index and total daily glycemic index with insulin, fasting glycemia, and HOMA-IR, in adults with obesity.

	Glycemia (<mg/dL)		Insulin (µU/mL)		HOMA-IR	
	β (95%CI)	p	*β* (95%CI)	p	*β* (95%CI)	p
**Dinner GI**						
Modelo I	-0.215 (-0.342, -0.087)	**0.001**	0.112 (0.013, 0.211)	**0.027**	0.014 (-0.011, 0.039)	0.280
Modelo II	-0.185 (-0.309, -0.061)	**0.004**	0.088 (-0.011, 0.187)	0.080	0.009 (-0.016, 0.034)	0.491
Modelo III	-0.174 (-0.300, -0.048)	**0.007**	0.090 (-0.003, 0.184)	0.058	0.010 (-0.014, 0.033)	0.415
Modelo IV	-0.172 (-0.298, -0.045)	**0.008**	0.084 (-0.009, 0.177)	0.077	0.008 (- 0.015, 0.032)	0.491
**Total daily GI**	*β* (95%CI)	p	*β* (95%CI)	p	*β* (95%CI)	p
Modelo I	-0.277 (-0.521, -0.033)	**0.026**	0.364 (0.184, 0.544)	**<0.001**	0.070 (0.024, 0.116)	**0.003**
Modelo II	-0.192 (-0.425, 0.042)	0.107	0.301 (0.122, 0.480)	**0.001**	0.058 (0.012, 0.104)	**0.013**
Modelo III	-0.241 (-0.481, -0.001)	**0.049**	0.280 (0.107, 0.452)	**0.002**	0.050 (0.006, 0.094)	**0.025**
Modelo IV	-0.240 (-0.484, 0.004)	**0.054**	0.302 (0.127, 0.477)	**0.001**	0.056 (0.012, 0.100)	**0.013**

GI: glycemic index. Model I: without adjustment. Model II: adjusted by age, sex, total energy intake, and BMI. Model III: adjusted by model II variables' and *FAAH C385A* variant. Model IV: adjusted by model II I variables and dinner protein

Figure S1. Heat map of Spearman's Rho related to different dietary and biochemical variables

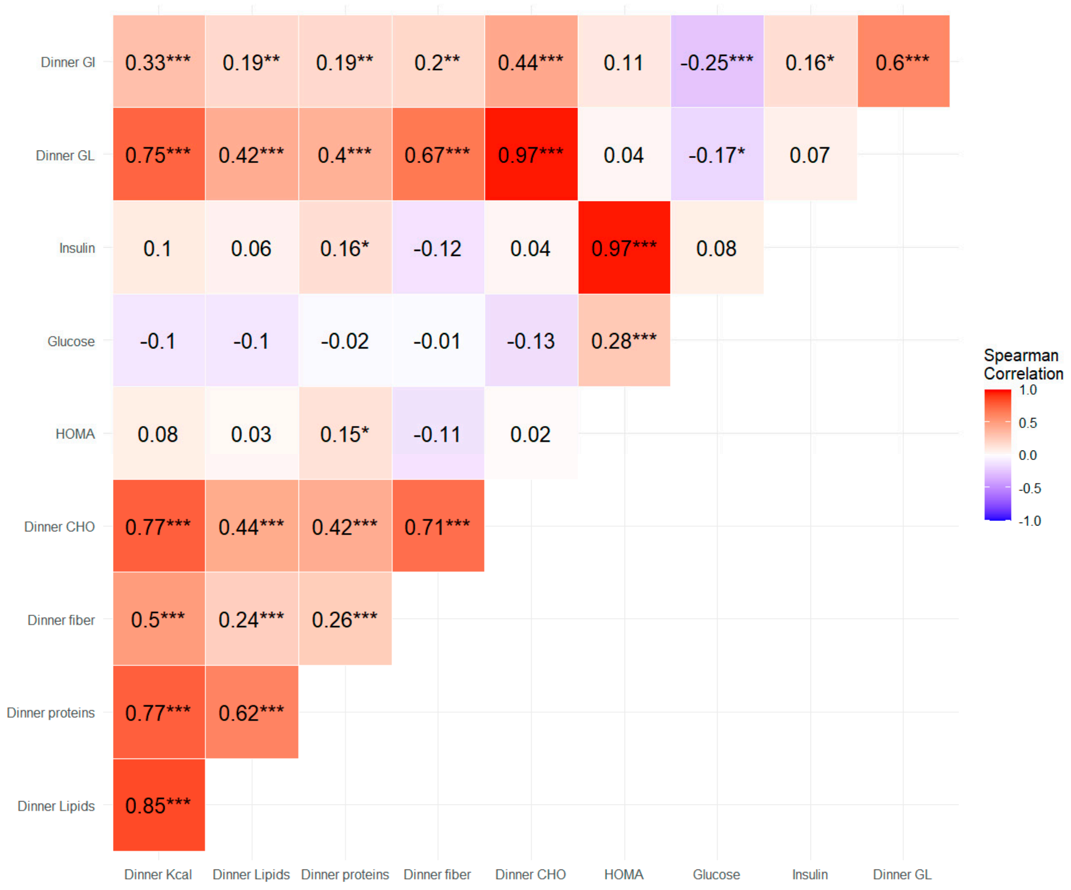

The heat map illustrates the correlations of dinner glycemic index (GI) and dinner glycemic load (GL) with insulin, glucose, HOMA-IR, dinner carbohydrates (CHO), fiber, and protein intake. Red indicates positive correlations and purple indicates negative correlations. Color intensity reflects the magnitude of the correlation coefficient. Statistical significance levels are denoted as ***$p < 0.001$, **$p < 0.01$, and *$p < 0.05$.
